# Flexible Nanocomposites Based on Polydimethylsiloxane Matrices with DNA-Modified Graphene Filler: Curing Behavior by Differential Scanning Calorimetry

**DOI:** 10.3390/polym12102301

**Published:** 2020-10-08

**Authors:** Elisa Toto, Susanna Laurenzi, Maria Gabriella Santonicola

**Affiliations:** 1 Department of Chemical Materials and Environmental Engineering, Sapienza University of Rome, Via del Castro Laurenziano 7, 00161 Rome, Italy; elisa.toto@uniroma1.it; 2 Department of Astronautical Electrical and Energy Engineering, Sapienza University of Rome, Via Salaria 851-881, 00138 Rome, Italy; susanna.laurenzi@uniroma1.it

**Keywords:** curing behavior, polysiloxanes, nanocomposites, graphene, calorimetry

## Abstract

Novel silicone-based nanocomposites with varied elastic properties were prepared by blending standard polydimethylsiloxane (PDMS) with a lower viscosity component (hydroxyl-terminated PDMS) and integrating a graphene nanoplatelets (GNP) filler modified by strands of deoxyribonucleic acid (DNA). The curing behavior of these nanocomposites was studied by dynamic and isothermal differential scanning calorimetry. The activation energies of the polymerization reactions were determined using the Kissinger method and two model-free isoconversional approaches, the Ozawa–Flynn–Wall and the Kissinger–Akahira–Sunose methods. Results show that the complex trend of the curing behavior can be described using the isoconversional methods, unveiling lower activation energies for the nanocomposites with standard PDMS matrices. The role of the DNA modification of graphene on the curing behavior is also demonstrated. The curing reactions of the nanocomposites with the PDMS matrix are favored by the presence of the GNP–DNA filler. PDMS/PDMS–OH blends generate softer nanocomposites with hardness and reduced elastic modulus that can be tuned by varying the amount of the filler.

## 1. Introduction

Composite materials consisting of carbon-based fillers embedded in an elastomeric polymer matrix are increasingly considered for stretchable and flexible electrodes in applications such as sensors, transducers and actuators in different fields, from aerospace technology to biomedicine and biotechnology [1,2,3]. In particular, the interest in silicone nanocomposites with graphene filler is steadily growing as they can be endowed with a wide array of multifunctional properties by integrating the exceptional mechanical strength and the good thermal and electrical conductivities of graphene nanoplatelets (GNP) [4,5,6] with the flexibility of a nontoxic polymer matrix, such as polydimethylsiloxane (PDMS), which is widely used in biocompatible materials and wearable devices [7,8].

In the design of graphene nanocomposites with tailored properties, the modification of the filler surface is often useful to enhance the integration of the filler with the polymer matrix or to add a novel functionality to the overall nanocomposite [9]. For example, the modification of GNP with DNA enables the creation of a UV-sensitive nanofiller, which can be used in radiation monitoring devices [10,11]. Due to its sensitivity to UV light [12], DNA can be used as a biological target for the detection of the UV-induced damage, and at the same time acts as a solubilizing and exfoliating agent for the graphene nanoplatelets in aqueous solutions. The DNA-modified graphene nanofiller was successfully embedded in a silicone (Sylgard 184 PDMS) matrix, which is transparent to UV radiation, thus creating a UV-sensitive stretchable nanocomposite [13]. In order to prevent the alteration of their electrical properties, GNPs can be functionalized by DNA molecules through non-covalent interactions, involving DNA functional moieties and oxidized groups on the GNP edges or base-graphene π stacking [14,15]. By varying the content of the DNA-modified graphene filler it is possible to obtain nanocomposites with different degrees of flexibility and electrical conductivity. In the biomedical field, this would allow for the use of such nanocomposites as soft elastic substrates for cell proliferation [16] or in devices that exploit their electro-mechanical properties, such as pressure sensors for artificial skin [17,18]. DNA-functionalized graphene nanostructures with different electrical properties can be developed by changing the type of non-covalent functionalization [19]. Botti et al. have also shown that the electrical behavior of GNP/DNA assemblies is affected by the DNA molecule orientation (tilted or flattened) with respect to the graphene surface, as revealed by surface-enhanced Raman spectroscopy [20].

The curing process of polymer matrices and how it is affected by the presence of fillers (modified or not) is a crucial aspect in the design and fabrication of nanocomposite materials [21]. In general, the specific properties of polymer-based composites are strictly related to the kinetics of the involved curing reactions [22,23]. In this perspective, differential scanning calorimetry (DSC) represents one of the most effective techniques to investigate the curing conditions of nanocomposites so to choose the most suitable process parameters for their fabrication [24,25,26]. The possibility to assess the filler effects on the curing behavior of polymer matrices allows to avoid using nanoparticles that can negatively affect the polymerization (in terms of high activation energies, long times or high temperatures required), and in some cases, detecting possible advantages such as an accelerating effect on the process [27,28]. A DSC analysis enables to determine the parameters that describe the curing phenomenon, which is denoted by a typical exothermic peak, such as enthalpy value, peak temperature and the corresponding cure time. Starting from the DSC data, the curing kinetics can be examined by estimating the activation energy values using different methodologies. In the case of filled polymer matrices and other complex polymer systems, many authors have relied on isoconversional methods [29,30,31,32] to have an assessment of the variation of the activation energy with the extent of cure. These methods, such as the Ozawa–Flynn–Wall (OFW) and Kissinger–Akahira–Sunose (KAS) ones, are based on the assumption that the reaction rate is only a function of the temperature. On the other hand, the Kissinger method represents a more simplistic method for the estimation of the activation energy [33]. Nevertheless, this method can be applied, as done by most of the authors, with the purpose to compare and eventually validate its outcome with respect to more complex approaches. 

The aim of this study was to investigate the curing behavior of silicone/GNP–DNA nanocomposites and the role of the DNA-modified GNP filler on the curing kinetics. In particular, we focused on nanocomposites with a PDMS-based matrix determining their activation energy profiles using differential scanning calorimetry under non-isothermal conditions. Different nanocomposite samples are investigated varying the type of matrix, PDMS (Sylgard 184) or its mixture with hydroxyl-terminated PDMS (PDMS–OH), and the amount of GNP–DNA filler. The use of a PDMS/PDMS–OH matrix (ratio 75:25 w/w) allowed enhancing the filler dispersion, by lowering the prepolymer viscosity, and also affects the elasticity of the cured nanocomposite [34]. The overall goal is to determine the curing kinetics of these types of nanocomposites, which adds valuable information for the materials processing and highlights possible limitations due to the presence of the filler.

## 2. Materials and Methods

### 2.1. Materials

Graphene nanoplatelets were purchased from XG Sciences (Lansing, MI, USA) and they were of grade C750, consisting of aggregates of platelets with a diameter of less than 2 µm, thickness of a few nm, and average surface area of 750 m^2^/g according to the manufacturer datasheet. Two-component polydimethylsiloxane (PDMS, Sylgard 184) was obtained from Dow Corning (Midland, MI, USA). Double-stranded DNA (product number 74782, purity ≤1% protein, A_260/280 nm_ ≥ 1.5) was purchased from Sigma-Aldrich (Milan, Italy) and used as received. Hydroxyl-terminated polydimethylsiloxane (PDMS–OH, average M_n_ ~550) was obtained from Sigma-Aldrich (Milan, Italy). Sterile deionized water (resistivity 18.2 MΩ·cm) was produced by a Direct-Q3 UV water purification system (Millipore, Molsheim, France) and used to prepare DNA solutions and DNA/graphene dispersions.

### 2.2. Preparation of Nanocomposite Mixtures

**Figure 1:**
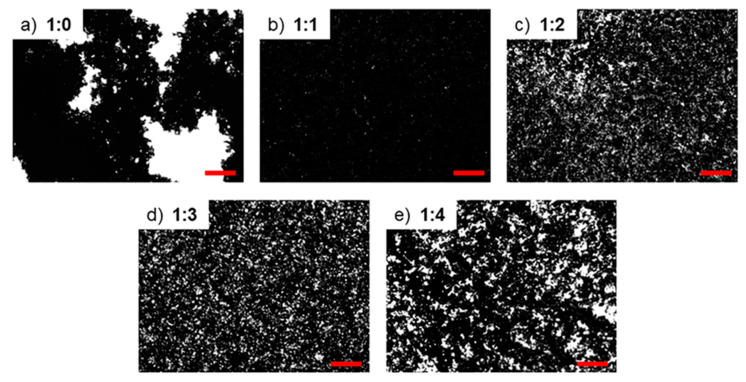
Optical images of aqueous dispersions with different graphene nanoplatelets (GNP):DNA ratios (w/w): (**a**) 1:0; (**b**) 1:1; (**c**) 1:2; (**d**) 1:3; (**e**) 1:4. Scale bar: 300 µm.

Nanocomposite mixtures were obtained by adding the DNA-modified graphene filler to the silicone prepolymer made of neat PDMS or PDMS/PDMS–OH (75:25 w/w) blend. The GNP–DNA filler was prepared by dispersing the GNP powder (grade C750) in an aqueous solution of DNA (2.5 mg/mL) and sonicating for 1 h in ultrasonic bath (Elmasonic P30H, Elma, Singen, Germany) with cold water to prevent DNA denaturation. Graphene and DNA were mixed in a ratio of 1:1 (w/w), which assures the best degree of dispersion of the nanoplatelets in the DNA solution. Figure 1 shows the GNP–DNA dispersions at different ratios (by weight) deposited on glass slides and imaged in transmitted light using a Leica polarized light microscope (DMLP series, Leica Microsystems, Wetzlar, Germany).

The successful non-covalent functionalization of the graphene filler by DNA without altering the nanoplatelet properties was demonstrated by our group using surface-enhanced Raman spectroscopy and high-resolution SEM [20]. The dispersion was completely evaporated by slow stirring at 50 °C and the desired amount of dried GNP–DNA added to the PDMS base or PDMS/PDMS–OH blend. The nanocomposite mixtures were sonicated for 1 h in the cold bath. Then, the curing agent was added in a ratio of base to curing agent of 10:1 (w/w) and gently mixed mechanically. Different nanocomposite samples were realized varying the amount of GNP–DNA filler (1 wt% and 5 wt%) for each of the two types of silicone matrix. The PDMS/PDMS–OH matrix was prepared by mixing the two components in the ratio of 75:25 (w/w), resulting in a less viscous matrix than neat PDMS due to the lower molecular weight of hydroxyl-terminated PDMS (M_n_ ~550). The ratio 75:25 was selected as an optimal ratio following our previous work on the dispersion of graphene nanofillers in silicone-based matrices, where it was demonstrated that mixed PDMS matrices with higher amounts of hydroxyl-terminated PDMS could not be successfully cured at the conditions used in this study [34].

### 2.3. DSC Experiments

The curing process of the nanocomposites was investigated by differential scanning calorimetry under non-isothermal conditions at heating rates of 5, 10, 15 and 20 °C/min. Isothermal scans were performed at T = 50 °C on selected PDMS-based samples. A Pyris 1 DSC instrument (Perkin–Elmer, Waltham, MA, USA) calibrated with high-purity indium and tin materials was used. DSC samples were prepared starting from each mixture immediately after the addition of the curing agent. Samples (~20 mg) were sealed in aluminum pans with lids and measured in the temperature range from 30 °C to 150 °C under a constant flow of nitrogen (20 mL/min). An identical empty cell was taken as the reference. A baseline was measured at the same experimental conditions using an empty cell and subtracted from the sample data. The exothermic peaks were analyzed using the thermal analysis software provided with the instrument. The heat flow data were processed to obtain the extent of conversion (α) as a function of temperature using the Perkin–Elmer Kinetics Software package (Perkin–Elmer, Waltham, MA, USA).

### 2.4. Kinetic Models for DSC Analysis

Data collected in the non-isothermal calorimetric experiments were implemented into different dynamic kinetic models, with the aim to estimate and compare the activation energy values related to the curing reactions. The first approach consisted in the application of the Kissinger method [33], which is based on the following equation:

(1) $$\ln\left(\frac{\beta }{T_{P}^{2}}\right)=\ln\left(\frac{\mathrm{AR}}{E}\right)\;-\;\frac{E}{{\mathrm{RT}}_{P}}$$

where β is the linear heating rate, T_p_ is the peak temperature, A and E are the pre-exponential factor and activation energy, respectively, and R is the universal gas constant. By plotting ln(β/T_p_^2^) versus 1/T_p_ it is possible to estimate the values of A and E. This method assumes that the maximum reaction rate occurs at peak temperature, corresponding to the exothermal peak position in the dynamic scan, and it exploits the correlation between this temperature value and the heating rate. One of the main limitations of the Kissinger method is the assignment of a single activation energy value to the entire process, which means that the assumption of a simple kinetic of reaction is based on a single step. In general, the Kissinger method fails when multiple reaction mechanisms are present or when the activation energy depends on the extent of conversion (α). For these cases, isoconversional methods have been developed [35,36], which require to determine the temperature (T_α_) associated with a certain extent of conversion for a fixed heating rate. These methods are based on the subdivision of the entire kinetic process into multiple single steps, where each step is characterized by a single value of the extent of conversion in a restricted range of temperature. Small variations of the activation energy values with the extent of conversion can legitimate the application of these methods, but without excluding other effects that can influence their use, as recommended by the Kinetics Committee of the International Confederation for Thermal Analysis and Calorimetry (ICTAC) [37]. The Ozawa–Flynn–Wall (OFW) method [38,39] is based on the equation:

(2) $$\ln\left(\beta_{i}\right)=\mathrm{const}-1.052\;\left(\frac{E_{\alpha }}{{\mathrm{RT}}_{\alpha }}\right)$$

whereas the Kissinger–Akahira–Sunose (KAS) method [40] relies on the equation:

(3) $$\ln\left(\frac{\beta_{i}}{T_{\alpha ,i}^{2}}\right)=\mathrm{const}-\;\left(\frac{E_{\alpha }}{{\mathrm{RT}}_{\alpha }}\right)$$

Here, the subscript α indicates isoconversional values, i.e., the values related to a given extent of conversion, and the index i identifies an individual heating rate (for non-isothermal programs). After studying the relation between the temperature values of the dynamic scan and the related extents of conversion, at each heating rate, the OFW and KAS methods were applied plotting ln(β_i_) versus 1/T_α_ and ln(β_i_/T_α_^2^) versus 1/T_α_, respectively. By comparing the results from the Kissinger and isoconversional approaches, it is possible to evaluate the consistency of the estimated activation energies. Since they are based on the same assumptions, the results from the two isoconversional methods (OFW and KAS) only differ in the degree of accuracy. In fact, the OFW and KAS equations were derived by the same linear equation, in the following general form [37]:

(4) $$\ln\left(\frac{\beta_{i}}{T_{\alpha ,i}^{B}}\right)=\mathrm{const}-C\;\left(\frac{E_{\alpha }}{{\mathrm{RT}}_{\alpha }}\right)$$

The parameters B and C were approximated to 0 and 1, respectively, by Doyle [41] obtaining the OFW equation (Equation (2)). Murray and White gave a more accurate approximation by setting B = 2 and C = 1 [37], which leads to the KAS equation (Equation (3)).

### 2.5. Characterization of Nanocomposites after Cure

Nanocomposite samples were prepared by casting method in circular molds (diameter of 35 mm, thickness of 5 mm), and they were cured in an oven at 50 °C for 24 h. Nanoindentation tests were performed using a NanoTest Platform instrument (Micro Materials Ltd., Wrexham, UK) with ten indentations in random positions on each specimen, with six repeats for each point. Experiments were performed with a spherical tip, under different applied loads (0.5 and 1 mN) with a loading rate of 0.5 mN/s and holding the maximum load for 20 s. Hardness and reduced elastic modulus were calculated from the load–displacement curves [42]. Optical images were acquired using a Leica polarized light microscope (DMLP series, Leica Microsystems, Wetzlar, Germany). Contact angles were measured by the sessile drop method using a DataPhysics OCA15Pro analyzer (DataPhysics Instruments, Filderstadt, Germany) with degassed ultrapure water as a testing liquid. A minimum of ten drops (volume 3 μL) on different areas of the substrate were analyzed for each nanocomposite sample. The determination of the water contact angle (WCA) values was performed according to the Young–Laplace fitting method using the DataPhysics SCA20 image analysis software (DataPhysics Instruments, Filderstadt, Germany). 

## 3. Results and Discussion

### 3.1. Calorimetry Analysis

**Figure 2:**
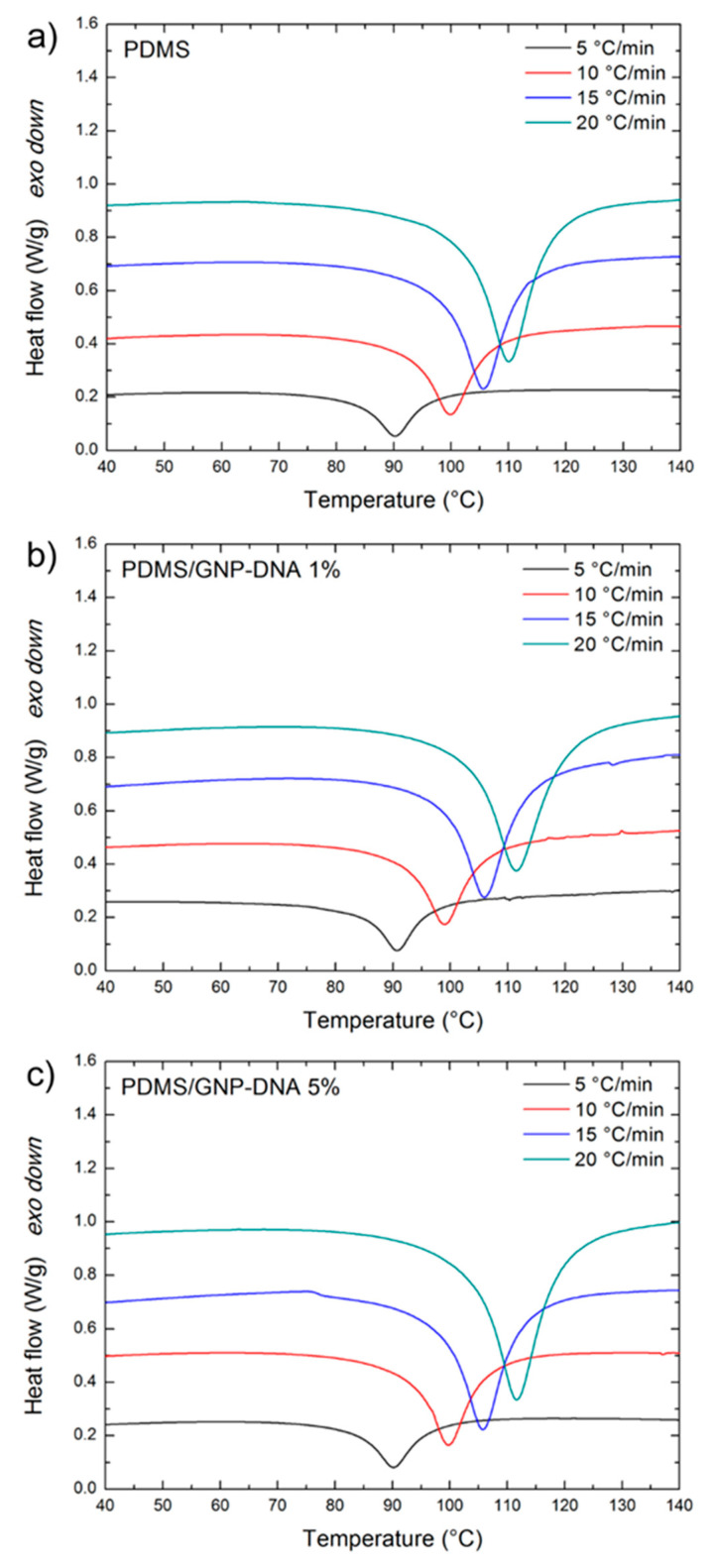
Differential scanning calorimetry (DSC) thermograms of (**a**) polydimethylsiloxane (PDMS) (Sylgard 184), (**b**) PDMS/GNP–DNA 1 wt% and (**c**) PDMS/GNP–DNA 5 wt% nanocomposite mixtures at different heating rates.

**Figure 3:**
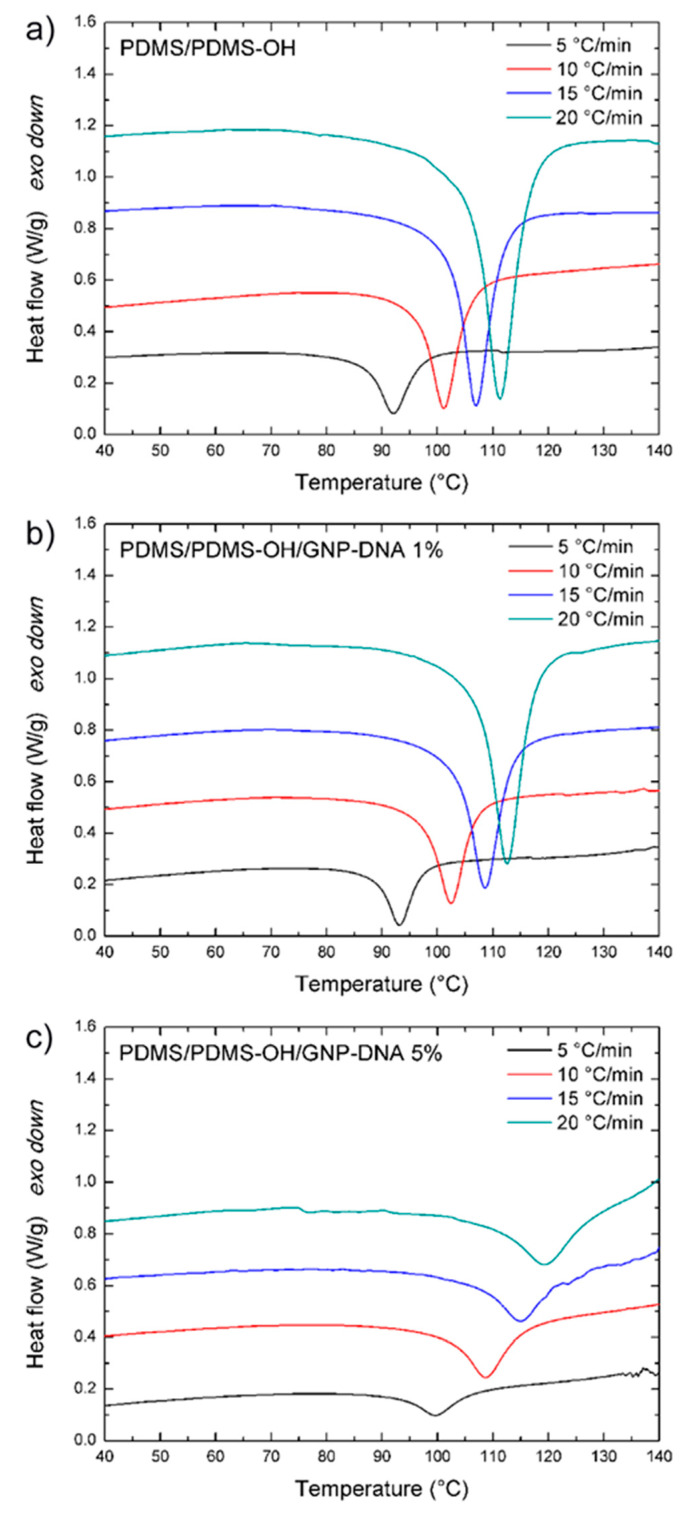
DSC thermograms of (**a**) PDMS/hydroxyl-terminated PDMS (PDMS–OH) (75:25 w/w), (**b**) PDMS/PDMS–OH/GNP–DNA 1 wt% and (**c**) PDMS/PDMS–OH/GNP–DNA 5 wt% nanocomposite mixtures at different heating rates.

Dynamic differential scanning calorimetry was used to analyze the curing kinetics of nanocomposite mixtures made of silicone-based matrices containing GNP nanofiller modified with DNA. First, the unfilled polymer matrices, Sylgard 184 PDMS and its blend with hydroxyl-terminated PDMS were investigated. Figure 2a and Figure 3a show the non-isothermal scans for the neat PDMS and PDMS/PDMS–OH systems, respectively, at the four heating rates considered in this study (5, 10, 15, 20 °C/min). The typical exothermic peaks of the PDMS curing reactions occur in the range from 90 to 110 °C and are slightly shifted to higher temperature (1–2 °C at most) for the PDMS/PDMS–OH blend. After the addition of the GNP–DNA nanofiller to the PDMS matrix (Figure 2b,c), the position and depth of the exothermic peak do not vary significantly with respect to the neat matrix (Figure 2a). A different situation is noted for the nanocomposites with the PDMS/PDMS–OH matrix, as a large shift of the peak combined with a decrease in the area under the curve can be observed (Figure 3b,c), particularly after the addition of 5 wt% of GNP–DNA. This result is a first indication of the different compatibility of the DNA-modified GNP nanofiller with the PDMS and PDMS/PDMS–OH matrices and will be investigated in more detail later, in terms of the activation energy of the curing process using different kinetic models. From the analysis of the exothermic peaks of the DSC thermograms, the onset, peak and end temperatures, and the heat of reaction (ΔH) were determined. These parameters for the unloaded matrices and for the nanocomposites with the GNP–DNA filler are summarized in Table 1 and Table 2. 

In general, for the standard PDMS (Sylgard 184), the characteristic temperatures of the exotherms show a relatively small increase, at fixed heating rate, upon the addition of the GNP–DNA filler. On the other hand, the curing parameters of the nanocomposites with the PDMS/PDMS–OH (75:25 w/w) matrix vary significantly with the amount of GNP–DNA filler. In fact, a shift of the exotherms to higher temperatures and a marked decrease in the heat of reaction values at higher concentrations of GNP–DNA are observed. This can be ascribed to a reduced compatibility between the filler and the hydroxylated component of the matrix, resulting in a delay in the curing of the PDMS/PDMS–OH blend in the presence of the GNP–DNA filler. This behavior is generally observed when the filler inhibits the polymer crosslinking process [43,44]. In particular, at the heating rate of 10 °C/min, there is an increase of 5.8–9.0% of the exotherm characteristic temperatures and a decrease of 25% of the heat of reaction, when comparing the unloaded PDMS/PDMS–OH matrix and the one filled with 5 wt% of GNP–DNA. It is interesting to note that, at each heating rate, the unloaded matrices show similar curing behavior in terms of peak temperature and the heat of reaction values. However, the onset temperature of the exotherms of the PDMS/PDMS–OH blend is always higher than that of the PDMS matrix.

When comparing the curing behavior of the nanocomposites with GNP–DNA filler, the situation is quite different depending on the type of silicone matrix. For the nanocomposites with the PDMS matrix, the characteristic temperatures and ΔH values of the exothermic peaks do not vary significantly upon the addition of the GNP–DNA filler. Samples containing 5 wt% of GNP–DNA show a difference of less than 1% in the exothermic peak temperature with respect to the empty PDMS matrix, and a difference of 5–7% in the ΔH values (Table 1). On the other hand, for the nanocomposites with PDMS/PDMS–OH (75:25 w/w) matrix, the curing behavior is strongly affected by the presence of the GNP–DNA filler. In particular, there is an increase of 7–8% in the peak temperatures and a decrease of 26–49% in the ΔH values (depending on the heating rate) for the nanocomposites with 5 wt% of GNP–DNA with respect to the unloaded PDMS/PDMS–OH blend (Table 2). These values indicate that the filler is more compatible with the neat PDMS matrix, whereas it affects negatively the curing kinetics of the nanocomposites with PDMS/PDMS–OH matrix.

Analysis of the trends of the characteristic temperatures (T_onset_, T_peak_, and T_end_) of the exothermic peaks only provides a qualitative assessment of the nanocomposites curing process and is not sufficient to thoroughly understand the dynamics of the curing reactions. The variation of enthalpy (ΔH), calculated as the area underneath the exothermic peak, adds important information to the analysis allowing to evaluate the extent of the conversion (α) of the reaction. The value of α can be calculated as the ratio of ΔH_i_, corresponding to a fixed degree of conversion i, and the overall variation of enthalpy of the curing reaction:

(5) $$\alpha_{i}=\frac{{\Delta H}_{i}}{\Delta H}$$

**Figure 4:**
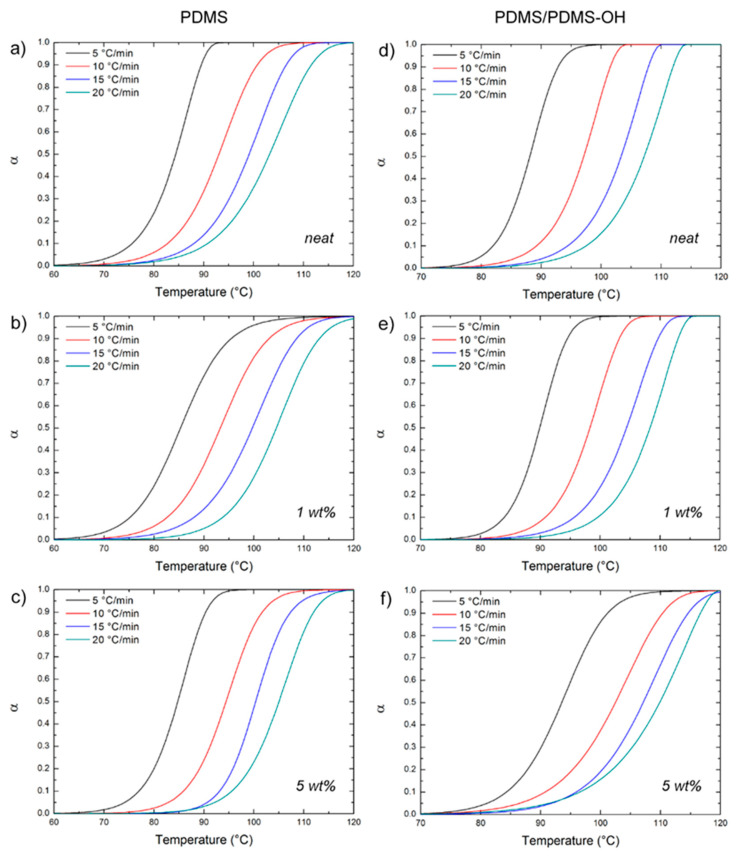
Extent of conversion (α) as a function of temperature for the nanocomposites with PDMS (Sylgard 184) and the PDMS/PDMS–OH (75:25 w/w) matrix at different heating rates: (**a**,**d**) unfilled; (**b**,**e**) filled with 1 wt% GNP–DNA; (**c**,**f**) filled with 5 wt% GNP–DNA.

For each sample made of neat silicone matrix or nanocomposite, the extent of conversion was plotted as a function of temperature at each fixed scan rate, obtaining the sigmoidal curves showed in Figure 4. Here, the sigmoidal shape is typical of an autocatalytic reaction mechanism, and its invariance upon the addition of the filler indicates that the kinetics of the curing process are not altered by the presence of the filler. This is more evident for the samples with a PDMS matrix (Figure 4a,c), whereas for the nanocomposites with the PDMS/PDMS–OH matrix, a shift in the sigmoidal towards higher temperatures is observed when increasing the content of the GNP–DNA filler (Figure 4d,f), suggesting a delay in the curing reaction caused by the filler addition.

**Figure 5:**
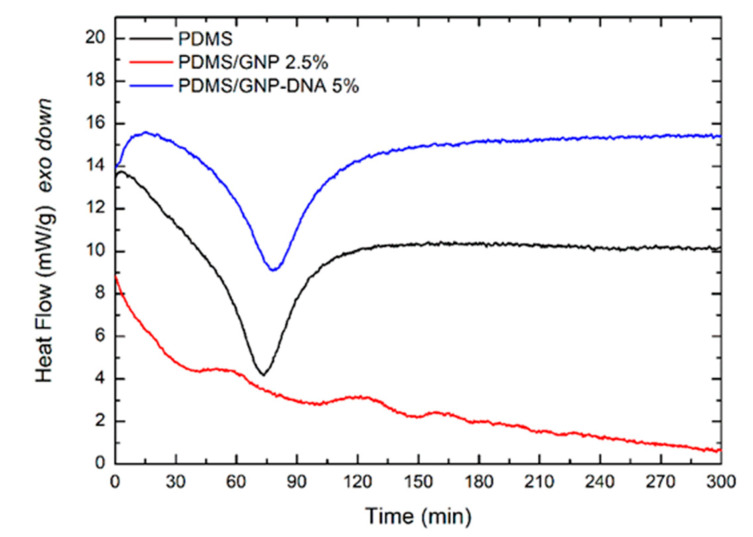
Isothermal DSC analysis (T = 50 °C) of the cure kinetics of PDMS prepolymer and PDMS nanocomposite mixtures filled with 2.5 wt% GNP and 5 wt% GNP–DNA.

Isothermal studies were conducted to investigate the role of DNA on the curing process of the PDMS-based nanocomposites. The experiments were carried out at T = 50 °C taking into account the thermal sensitivity of DNA, which degrades at temperatures higher than 90 °C. In addition, since the mechanical properties of elastomers are known to vary with curing temperature [45], we used a lower temperature in order to obtain soft and flexible materials suitable for bioelectronics applications. PDMS prepolymer and PDMS nanocomposite mixtures filled with the same weight percentage of GNP (2.5 wt%) (Figure 5) were examined. Results unveiled that the presence of DNA strongly favors the curing process of the mixtures, and curing does not occur when unmodified GNP powder (grade C750) is used. 

### 3.2. Modeling of the Nanocomposites Curing Reactions

Starting from the data collected in the dynamic calorimetric experiments, different kinetic models were implemented with the aim to estimate and compare the activation energy values of the curing reactions. First, the Kissinger method was applied by plotting ln(β/T_p_^2^) versus 1/T_p_, using the values of peak temperature extracted from the thermograms at different heating rates (Table 1 and Table 2). For all the cases of nanocomposite systems (with the PDMS and PDMS/PDMS–OH matrix), the Kissinger equation gave a linear relationship with a high correlation factor (R-squared > 0.9884) and the curing activation energy (E) was determined confidently from the slope (Equation (1)). Results from the linear regression are reported in Table 3. By comparing the curing activation energies of the PDMS and PDMS/PDMS–OH neat matrices, it was observed that the value of E was slightly higher for the mixed silicone system. This can be explained by the presence of the hydroxyl-terminated PDMS, which slows down the curing reaction of PDMS (Sylgard 184) occurring by cross-linking at the vinyl groups of the base component. Nevertheless, nanocomposites with a PDMS/PDMS–OH matrix at the ratio of 75:25 w/w can be successfully cured also at low temperatures (50 °C for 24 h), whereas mixed matrices with a higher content of hydroxyl-terminated PDMS (50:50 w/w) are only partially cured at the same conditions [34].

When considering the nanocomposite systems with the GNP–DNA nanofiller at increasing concentrations, we observed an opposite trend for the curing activation energy depending on the type of silicone matrix. For the nanocomposites with the PDMS (Sylgard 184) matrix, the presence of the filler contributes to lower the curing activation energy of the neat silicone, whereas when the matrix is made of the PDMS/PDMS–OH blend, the presence of the filler increases the activation energy. In particular, for nanocomposites with 5 wt% of GNP–DNA, the curing activation energy of the PDMS/PDMS–OH system is 13.2% higher than the nanocomposite with the PDMS matrix. This result points to the different interaction of the filler with the PDMS-based matrices, suggesting that the GNP–DNA assembly is less compatible with the hydroxyl-terminated PDMS component of the mixed silicone matrix, than with the neat PDMS (Sylgard 184).

**Figure 6:**
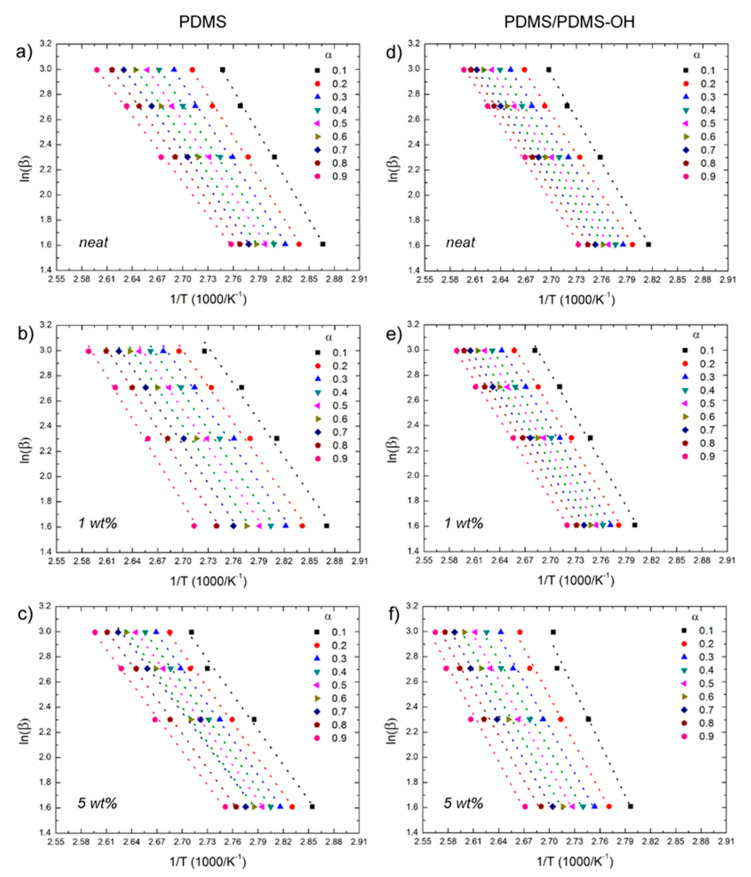
Plots of ln(β) versus 1/T at different extents of conversion according to the Ozawa–Flynn–Wall model for nanocomposites with the PDMS and PDMS/PDMS–OH (75:25 w/w) matrix: (**a**,**d**) unfilled; (**b**,**e**) filled with 1 wt% GNP–DNA; (**c**,**f**) filled with 5 wt% GNP–DNA.

**Figure 7:**
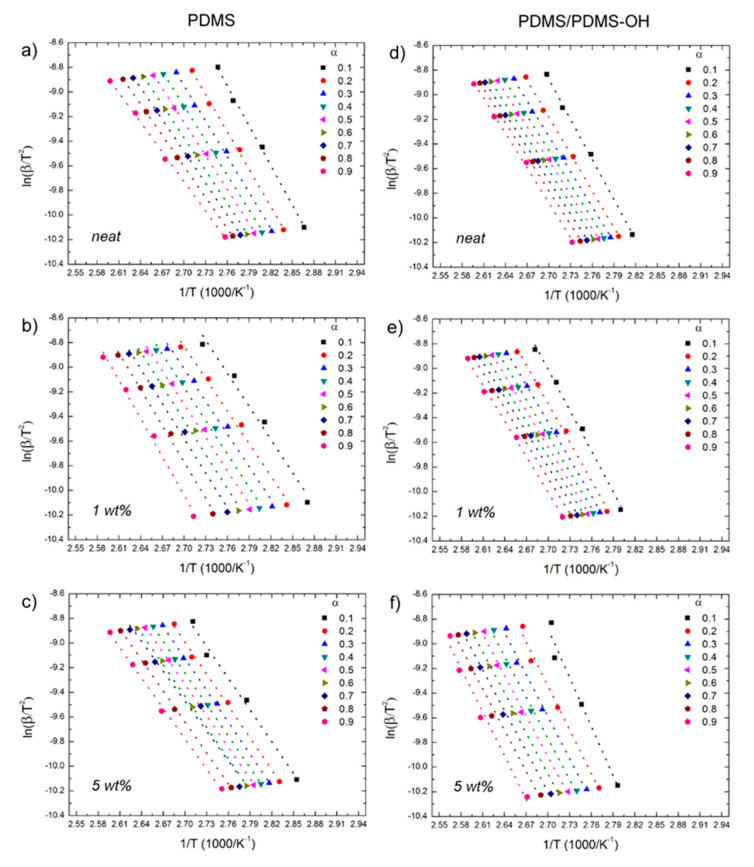
Plots of ln(β/T^2^) versus 1/T at different extents of conversion according to the Kissinger–Akahira–Sunose model for nanocomposites with the PDMS and PDMS/PDMS–OH (75:25 w/w) matrix: (**a**,**d**) unfilled; (**b**,**e**) filled with 1 wt% GNP–DNA; (**c**,**f**) filled with 5 wt% GNP–DNA.

**Figure 8:**
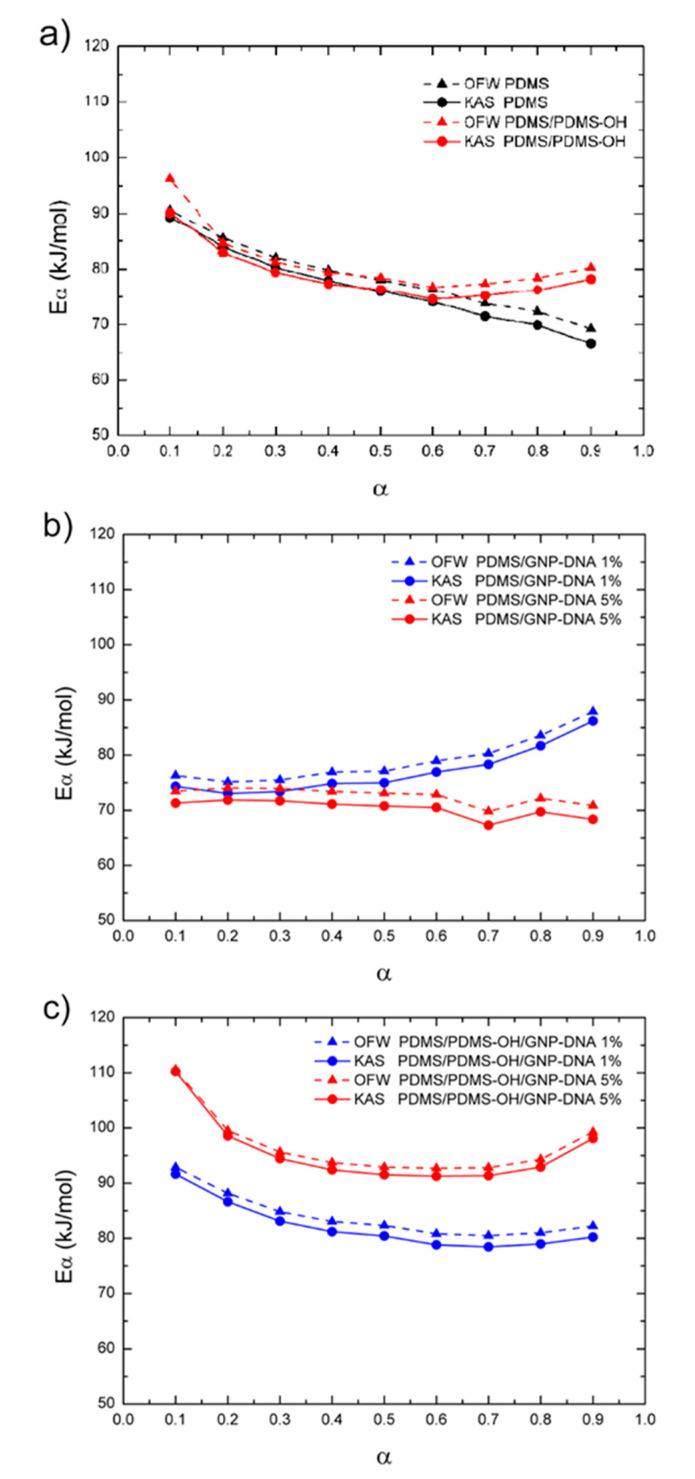
Activation energies (E_α_) of the curing process of (**a**) the unfilled PDMS matrix and PDMS/PDMS–OH blend (75:25 w/w); (**b**) the PDMS nanocomposites filled with GNP–DNA (1 wt%, 5 wt%); (**c**) PDMS/PDMS–OH nanocomposites filled with GNP–DNA (1 wt%, 5 wt%) estimated by Ozawa–Flynn–Wall (OFW) and Kissinger–Akahira–Sunose (KAS) methods.

After a first assessment of the activation energy by the Kissinger method, the curing process of the elastomeric nanocomposites was analyzed using the OFW and KAS isoconversional methods, following Equation (2) and Equation (3), respectively, and using the experimental data on the extent of conversion (α) as a function of temperature that are reported in Figure 4. Plots of ln(β) versus 1/T (Figure 6) and of ln(β/T^2^) versus 1/T (Figure 7) were generated for the implementation of the OFW and KAS methods, respectively. Each data set was plotted at a fixed extent of conversion (α) ranging from 0.1 to 0.9, and the value of the activation energy (E_α_) was estimated from the slope of the linear fit. Results from the OFW and KAS analyses, in terms of the activation energy of the curing process as a function of the extent of conversion α, are reported in Figure 8. Since the difference between the maximum and minimum values of E_α_ across the range of the conversion degree does not exceed 20–30% of its average [37], from the OFW and KAS approach, a mean value of E_α_ was calculated by averaging the values at each extent of conversion. Results are summarized in Table 3, where it can be noted that there is a small difference between the mean values of E_α_ obtained from the two methods. Such a small difference has been reported by several authors [46,47,48], and is due to the different degree of accuracy of the two mathematical approaches. All methods are in good agreement as far as the curing behavior of the unfilled matrices is analyzed, and they all correctly indicate that the activation energy of the PDMS/PDMS–OH blend is higher than that of the neat PDMS matrix due to the presence of the silicone oil component. In general, the E_α_ values determined by the OFW and KAS methods are higher than those obtained by the Kissinger method, and most notably they show a larger variation for the nanocomposite samples with respect to the unfilled matrices. In fact, results by the Kissinger method do not show significant differences between the unfilled matrix and the nanocomposites, unlike what can be observed experimentally after the curing cycle, thus underestimating the effect of the nanofiller on the curing behavior of the PDMS-based matrices. In addition, the OFW and KAS approaches both confirm the opposite trend that the GNP–DNA nanofiller has on the curing kinetics of the two different matrices (Figure 8b,c). In the case of the PDMS matrix, the presence of the nanofiller causes a decrease in the activation energies, reaching an 8% decrease upon the addition of 5 wt% of GNP–DNA (Table 3). This can be explained by the high thermal conductivity of the graphene nanoplatelets [49,50], which enhances the cure kinetics of the PDMS when a sufficient amount of GNP is added. On the other hand, for the PDMS/PDMS–OH blend, a progressive and significant increase in the E_α_ values is observed upon addition of the GNP–DNA filler, with an increase of about 20% at 5 wt% of filler (Table 3). This result agrees with the experimental values of enthalpies determined by the DSC thermograms and reported in Table 2, indicating a lower degree of curing for the PDMS/PDMS–OH blend upon the addition of the nanofiller, and is due to the incompatibility of the hydrophobic GNP with the hydroxyl-terminated component (PDMS–OH) of the matrix. Overall, the results above indicate that the Kissinger method underestimates the presence of the filler in the elastomeric matrices, whereas the OFW and KAS isoconversional methods are more suitable for the analysis of the curing kinetics of these nanocomposite samples.

### 3.3. Mechanical Properties and Analysis of Surface Wettability

**Figure 9:**
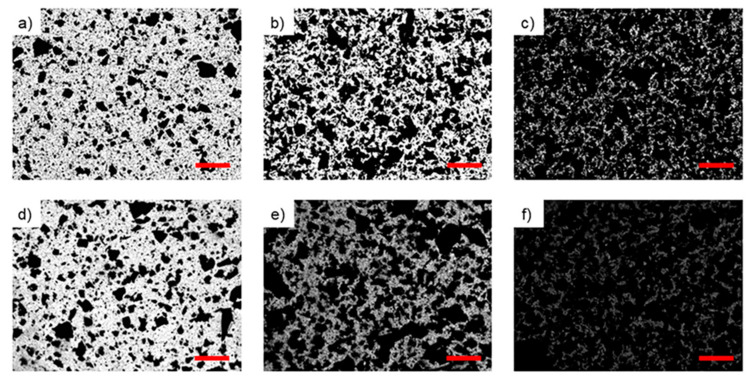
Optical images of nanocomposite samples fabricated by mold casting and cured at 50 °C for 24 h. Top: PDMS-based nanocomposites filled with (**a**) 1 wt%; (**b**) 3 wt%; and (**c**) 5 wt% of GNP–DNA. Bottom: PDMS/PDMS–OH-based nanocomposites filled with (**d**) 1 wt%; (**e**) 3 wt%; and (**f**) 5 wt% of GNP–DNA. Scale bar: 1 mm.

Following the calorimetric analysis of the cure behavior, the local surface mechanical properties of the elastomeric nanocomposites prepared from the two silicone matrices were determined by nanoindentation. Samples were cured for 24 h at 50 °C because of the thermal sensitivity of DNA, which undergoes degradation at temperatures higher than 90 °C. These parameters still ensure a complete cure of the nanocomposite mixtures as determined by post-cure DSC analysis resulting in the absence of any residual exothermic peak. In addition, it is known that PDMS can be cured at different temperatures in the range from ambient to 200 °C, obtaining substrates with different mechanical properties [45]. The morphology of the silicone-based nanocomposites after cure is shown in Figure 9. The optical images reveal a similar degree of dispersion of the GNP–DNA filler for both PDMS and PDMS/PDMS–OH matrices for filler loadings up to 5 wt%. 

**Figure 10:**
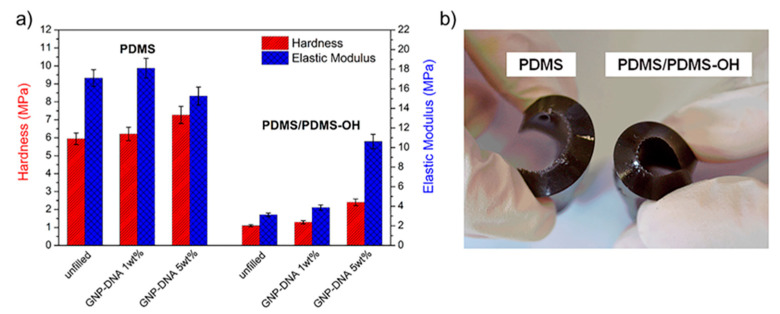
(**a**) Mechanical properties of nanocomposites with silicone-based matrices, the PDMS (Sylgard 184) and the PDMS/PDMS–OH blend (75:25 w/w), and GNP–DNA filler from nanoindentation tests (0.5 mN applied load, standard deviations in the range of 5–8%); (**b**) the flexibility of nanocomposites with 5 wt% of GNP–DNA.

The mean values of the hardness and reduced elastic modulus obtained by nanoindentation tests at 0.5 mN applied load are shown in Figure 10a. Results clearly indicate that the elastic behavior and the hardness of the two nanocomposite systems, with the PDMS and PDMS/PDMS–OH matrices, are quite different. The nanocomposites with the mixed PDMS/PDMS–OH matrix are much softer and with a reduced elastic modulus which varies significantly as a function of the nanofiller concentration. In contrast, the nanocomposite samples with the PDMS matrix show a quite similar elastic modulus, up to 5 wt% of GNP–DNA. In both cases, the material hardness tends to increase upon the addition of the modified graphene nanofiller. Figure 10b shows the different flexibility of the nanocomposites with PDMS and PDMS/PDMS–OH (75:25 w/w) matrices containing 5 wt% of GNP–DNA filler. 

**Figure 11:**
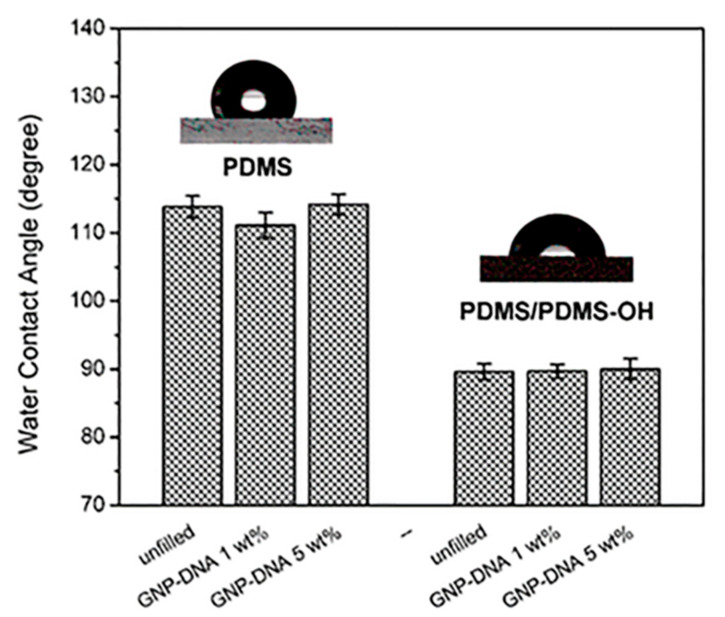
Comparison of the surface hydrophobicity of the standard PDMS (Sylgard 184) and the PDMS/PDMS–OH blend (75:25 w/w), unfilled and after the addition of 1 wt% and 5 wt% of GNP–DNA, as determined by the sessile drop method.

Finally, the surface wettability of the flexible nanocomposites was assessed by contact angle measurements at room temperature using the sessile drop method. A comparison of the water contact angles of the nanocomposites with PDMS and PDMS/PDMS–OH (75:25 w/w) matrices was reported in Figure 11. The addition of the less viscous PDMS–OH component to the silicone matrix significantly lowers its hydrophobicity due to the presence of the hydroxyl functional groups. The WCA value decreases from 113.8° ± 1.6° for the standard PDMS substrate to 89.6° ± 0.9° for the softer PDMS/PDMS–OH sample. No statistically relevant variation can be detected in the nanocomposites with respect to the unfilled matrices up to 5 wt% of the GNP–DNA filler. 

## 4. Conclusions

Polymer nanocomposites with silicone matrices filled with DNA-functionalized graphene were investigated by dynamic calorimetry to determine the curing reaction kinetics. The Kissinger method underestimates the presence of the filler in the elastomeric matrices, whereas the two isoconversional models (OFW and KAS) are able to follow the complex trend of the curing behavior of the nanocomposites as a function of the filler content. For a fixed filler content, the nanocomposites with standard PDMS are characterized by lower activation energies for the polymerization than those with the PDMS/PDMS–OH blend. The PDMS curing reactions are favored by the presence of the GNP–DNA filler, at sufficiently high content (5 wt%). Isothermal DSC experiments unveiled that the DNA component favors the curing process, as nanocomposites filled with pristine GNP do not cure. Nanoindentation tests showed that the cured nanocomposites with the PDMS/PDMS–OH matrix are softer than those with standard PDMS. The hardness and reduced elastic modulus can be tuned by varying the amount of the filler. Our work provides a facile and effective way to prepare silicone-based nanocomposites with varied flexibility and surface hydrophobicity.

## Figures and Tables

**Table 1:** Curing process parameters for PDMS (Sylgard 184) and its nanocomposites with GNP–DNA filler from DSC dynamic scans at different heating rates.

Sample	β (°C/min)	T_onset_ (°C)	T_peak_ (°C)	T_end_ (°C)	ΔH (J/g)
PDMS	5 10 15 20	83.3 91.9 98.0 101.8	90.2 99.3 105.7 110.2	96.4 106.7 112.6 117.8	22.0 22.6 24.1 25.2
PDMS/ GNP–DNA 1 wt%	5 10 15 20	83.9 92.0 98.2 103.0	90.7 99.0 105.9 111.5	96.8 106.0 113.4 120.7	23.9 21.6 22.9 24.3
PDMS/ GNP–DNA 5 wt%	5 10 15 20	83.5 93.4 98.2 103.7	90.2 99.8 105.7 111.5	96.6 106.4 112.7 119.6	23.7 24.3 24.5 26.6

**Table 2:** Curing process parameters for the PDMS/PDMS–OH (75:25 w/w) blend and its nanocomposites with GNP–DNA filler from DSC dynamic scans at different heating rates.

Sample	β (°C/min)	T_onset_ (°C)	T_peak_ (°C)	T_end_ (°C)	ΔH (J/g)
PDMS/PDMS–OH	5 10 15 20	86.9 96.1 102.2 106.1	92.0 101.1 107.1 111.3	97.2 105.9 112.0 116.8	22.5 23.6 24.6 24.5
PDMS/PDMS–OH/ GNP–DNA 1 wt%	5 10 15 20	89.2 97.4 103.4 107.4	93.2 102.5 108.6 112.7	97.1 107 113.5 117.6	20.6 20.4 20.7 22.6
PDMS/PDMS–OH/ GNP–DNA 5 wt%	5 10 15 20	92.2 101.7 106.0 109.0	99.7 108.7 115.1 119.3	107.1 115.5 112.9 128.3	16.6 17.7 13.4 12.4

**Table 3:** Activation energies for the curing process of PDMS (Sylgard 184) and PDMS/PDMS–OH (75:25 w/w) nanocomposite systems estimated by the Kissinger method (with R-squared values from linear regression) and the isoconversional methods, Ozawa–Flynn–Wall (OFW) and Kissinger–Akahira–Sunose (KAS).

Sample	E (kJ/mol)		
	Kissinger	OFW	KAS
PDMS	74.2 (0.9987)	78.6 ± 6.3	76.6 ± 6.7
PDMS/GNP–DNA 1 wt%	71.2 (0.9884)	79.1 ± 4.0	77.1 ± 4.1
PDMS/GNP–DNA 5 wt%	70.4 (0.9956)	72.6 ± 1.4	70.3 ± 1.5
PDMS/PDMS–OH	77.5 (0.9996)	81.4 ± 5.7	78.9 ± 4.6
PDMS/PDMS–OH/GNP–DNA 1 wt%	77.0 (0.9997)	84.0 ± 3.9	82.2 ± 4.1
PDMS/PDMS–OH/GNP–DNA 5 wt%	79.7 (0.9989)	96.8 ± 5.5	95.6 ± 5.8
